# Editorial: Beauty and the mind: cognitive science of the sublime

**DOI:** 10.3389/fpsyg.2025.1733274

**Published:** 2025-11-25

**Authors:** Claudio Lucchiari, Maria Elide Vanutelli, Fernando Echarri

**Affiliations:** 1 Department of Philosophy “Piero Martinetti”, Università degli Studi di Milano, Milan, Italy; 2 Department of Psychology, University of Milan-Bicocca, Milan, Italy; 3 University of Navarra Museum, Pamplona, Spain; 4 University of Navarra, Pamplona, Spain

**Keywords:** beauty, sublime, neuroscience, psychology, aesthetics

The concept of the sublime marks a pivotal stage in the evolution of modern aesthetics. Since the early works of Burke (1999) and Kant (1790), it has been described as a constellation of sensations emerging from the encounter between an individual and an object or environment whose magnitude, complexity, or power exceeds human comprehension. This confrontation with the limits of understanding generates a paradoxical pleasure—one that can arise from beauty, awe, or even fear.

Although closely related to beauty, the sublime engages distinct neural and phenomenological pathways. Beauty typically activates regions associated with reward and pleasure, such as the orbitofrontal cortex (Ishizu and Zeki, 2014), producing satisfaction rooted in perceptual harmony or personal association. The sublime, by contrast, evokes awe, vastness, and transcendence—emotions that elude straightforward explanation. Its pleasure lies not in balance, but in cognitive and emotional expansion before something perceived as greater than oneself.

Being a multifaceted phenomenon, the study of the sublime deserves renewed theoretical and empirical attention. This Research Topic aims to gather diverse perspectives to understand how beauty and the sublime shape human potential, and to explore its possible applications for psychological and cognitive wellbeing.

In contemporary psychology, the sublime has increasingly been reinterpreted not merely as an aesthetic judgment, but as a lived, embodied, and meaning-making experience. Extending this line of thought, Ding and Tan propose a culturally grounded and spiritually attuned model of sublime beauty rooted in Chinese philosophical traditions. Drawing on Confucian, Daoist, and Buddhist sources, they present a three-dimensional framework—potential, process, and goal—to explain how individuals realize “ideal humanity” through encounters with the sublime. Through examples from classical Chinese painters, the authors show how aesthetic practice simultaneously cultivates moral, cognitive, and spiritual dimensions of the self. Their model integrates art, cognition, and spirituality within cultural psychology, illustrating how sublime beauty may serve as a pathway toward human flourishing and creative consciousness.

While philosophical approaches have traditionally linked the sublime to human finitude, psychological research seeks to anchor it in lived experience, exploring its cognitive, emotional, and behavioral consequences. Recent studies demonstrate that specific aesthetic configurations—such as architectural symmetry—can elicit a subtle sense of harmony that promotes prosocial tendencies, as if perceivers momentarily attune to a larger order (Pizzolante et al., 2025). This line of inquiry suggests that the sublime operates largely below the threshold of conscious awareness, emerging when perceptual and emotional systems achieve a fleeting coherence. Neurocognitive models attribute this integrative capacity to the hippocampus, which synthesizes explicit and implicit information to create unified perceptual experiences (Danckert et al., 2007; Ishizu and Zeki, 2014).

Building on these foundations, Kudahl offer a phenomenological investigation of how architecture evokes the sublime. Through literary depictions by Woolf, Neruda, and Miller, they explore the embodied, affective, and atmospheric dimensions of built space. Their Husserlian approach reveals how elevation, solitude, and images of suffering can converge into moments of existential transformation—an aesthetic awakening that transcends mortality. By reading architectural experiences as lived encounters rather than symbolic representations, the study redefines the sublime as a psychological and spatial phenomenon. In doing so, it underscores how architectural atmospheres shape consciousness and emotion through multisensory, bodily participation, reinforcing the notion that sublime experience emerges through perceptual integration and embodied attunement.

At this regard, Akounach et al. advance the field by adopting a neuroscientific and embodied perspective on aesthetic experience. Using posturography, they examined how pleasant versus neutral natural scenes modulate both motor and affective responses. Participants viewed these landscapes under passive and active (embodied) conditions while postural and electrodermal activity were recorded. Subtle yet consistent postural shifts indicated approach tendencies toward pleasant scenes, especially under active viewing. These findings highlight the interaction between emotional valence, embodiment, and motor control, suggesting that immersion and mental simulation play a key role in aesthetic appreciation—an embodied echo of the sublime's capacity to unite cognition, emotion, and bodily engagement.

Psychological and neuroscientific studies have largely refuted strong associations between the sublime and negative emotions such as fear. Rather, the sublime appears as a distinct state of pleasurable self-transcendence, deeply subjective and unique to the perceiver. Echoing Kant's view that the sublime resides not in objects but in the mind, empirical research must thus rely on first- person reports to capture when and how the sublime truly occurs.

Within this perspective, two additional contributions highlight the subjective and psychological dimensions of sublime experience. First, Echarri et al. examined aesthetic sensitivity as an embodied disposition, focusing on how highly sensitive individuals engage more deeply with artistic and emotional stimuli. Integrating psychology, phenomenology, and art theory, the authors analyze how the paintings of Maselli and Rothko evoke immersive and transcendent states that blur the boundary between perception and emotion. They also introduced a new assessment tool for the perception of the sublime, aiming to operationalize this elusive construct. Their findings suggest that beauty functions as a relational and transformative process, particularly salient for individuals with heightened sensory and emotional awareness, thereby advancing empirical aesthetics and theories of aesthetic consciousness.

Second, Liu et al. address a timely psychological concern—self-objectification among women—through the lens of aesthetic and sociocultural pressures. They propose a model describing how the internalization of appearance norms, reinforced by social and media influences, fosters body surveillance, shame, and diminished self-concept clarity. This process mirrors the alienation from one's embodied self, opposing the integrative experience of the sublime. The authors identify self- concept clarity as a key vulnerability factor and suggest that interventions grounded in mindfulness and self-compassion could mitigate these maladaptive effects. By linking aesthetic ideals with psychological wellbeing, their framework bridges the gap between empirical aesthetics and clinical psychology.

Taken together, these contributions illustrate the interdisciplinary richness of the psychology of the sublime. Understanding how it operates may illuminate the processes through which humans construct meaning, experience connection, and cultivate wellbeing. Future research, integrating aesthetic science with neuroscience, phenomenology, and moral philosophy, promises to expand this field toward a comprehensive theory of human flourishing through the experience of the sublime.
